# Wearable energy harvesters generating electricity from low-frequency human limb movement

**DOI:** 10.1038/s41378-018-0024-3

**Published:** 2018-09-10

**Authors:** Keli Li, Qisheng He, Jiachou Wang, Zhiguo Zhou, Xinxin Li

**Affiliations:** 1 College of Life and Environmental Sciences, Shanghai Normal University, 200234 Shanghai, China; 2 State Key Laboratory of Transducer Technology, Shanghai Institute of Microsystem and Information Technology, Chinese Academy of Sciences, 200050 Shanghai, China; 3 The University of Chinese Academy of Sciences, 100049 Beijing, China

## Abstract

A wearable energy harvester technology is developed for generating electricity from the movement of human joints. A micro-electroplated ferromagnetic nickel cantilever is integrated with a piezoelectric element and bonded on a flexible substrate. Based on the magnetic interaction between the magnetized cantilever and a magnet on the substrate, a novel vertical-vibration frequency-up-conversion (FUC) structure is formed to generate stable amounts of electric energy per cycle from the horizontal substrate stretching/rebounding. The two ends of the flexible substrate are attached on both sides of a limb joint to transform joint rotation into substrate stretching. During limb movement, the flexible substrate is horizontally stretched and rebounded, causing the cantilever to vertically release from and return to the magnet, thereby exciting the piezoelectric cantilever into resonant generation. Since the horizontal low-frequency limb movement is perpendicular to the vertical high-frequency resonance, the stretch has little influence on the resonance of the cantilever. Thus the generated energy is always stable within a wide frequency range of limb movements. The performance of the novel harvester is experimentally verified using a stretching/rebounding movement cycle, where the cycle corresponds to the frequency range of 0.5–5.0 Hz. Within one stretching/rebounding movement cycle, the generated electric energy is stable in the approximate range of 0.56–0.69 μJ for the whole frequency range. Two flexible harvesters are worn on the human elbow and knee for a body kinetic energy harvesting test. Considerable power can always be generated under typical low-frequency limb movements, such as squatting, walking, jogging, and fast running, where the peak-to-peak generated voltages are always approximately 4.0 V. Additionally, energy harvesting under two-directional area stretching is also realized by adjusting the FUC structure layout. The flexible-substrate harvester is promising for various wearable applications.

## Energy generation: Harnessing people power

A wearable generator developed by researchers in China can convert the movement of arms and legs into useful electricity. A challenge for portable ‘kinetic harvesters’ based on piezoelectric technologies is that small devices have high resonant frequencies, whereas the frequency at which people move their limbs is low. Xinxin Li and co-workers at Shanghai Normal University and the Chinese Academy of Sciences solved this problem through an ingenious design using a nickel cantilever and a magnet, on a flexible base that can be stuck on a person’s arm or leg joint. When the base is stretched and squashed by bending a knee or elbow, the cantilever springs off and onto the magnet, each time resonating at high frequencies. The resonance produces voltages that could prove useful for healthcare, athletics and military applications.

## Introduction

Wearable devices are highly demanded for various microsystem applications, such as health care, medical rehabilitation, athletic training, and outdoor equipment^1,2^. Conventional wearable devices are mainly powered by batteries and thus have a limited working time period. Energy renewal or battery recharge for the devices is too inconvenient to satisfy “plug and play”^3,4^. To address this issue, the self-powered scheme, in which the device’s power is supplied by an attached wearable energy harvester, is increasingly attracting attention^5^. The basis of such a self-powered scheme lies in the fact that the human body contains abundant kinetic energy sources during limb movements, such as joint rotation^6–9^.

The conversion from kinetic energy to electric energy can be accomplished via piezoelectric, electromagnetic, and electrostatic effects^10–12^. Since conventional kinetic harvesters normally utilize resonant structures, e.g., cantilevers, only movements or vibrations near the resonant frequency can be efficiently harvested, thus hindering operation for a wide frequency band^13,14^. Many technical efforts have been made to broaden the frequency band, such as the use of multimode coupling, bi-stable structures, and stoppers^15–17^. However, wearable harvesters are normally miniature in size and feature a much higher frequency response of hundreds of Hz than the sub-Hz to several Hz slow-speed movement of human limbs^18^. Therefore, it is necessary to find a way, e.g., frequency-up-conversion (FUC), to bridge the great frequency disparity^19,20^. Galchev et al. proposed an interesting parametric frequency-increased generator for FUC generation under low-frequency vibrations^21^. The device consists of a large inertial mass and two generator structures placed on either side of the inertial mass for high-frequency oscillation. The inertial mass snaps back and forth between the two generator structures due to a magnetic attracting force. As the force on the inertial mass overwhelms the holding magnetic force, the inertial mass detaches, and the generator structures can generate electric energy. When the vibration amplitude is larger than the size of the device, the FUC scheme helps to break through the vibration displacement limit of the device. Hence, the FUC structure realizes more effective electric generation than conventional linear harvesters. Takahashi et al. proposed another kind of attraction-based FUC energy harvester in which an electrostatic attracting force is used for frequency conversion^22^. Additionally, impact-based FUC harvesters have been reported, which usually consist of sliding balls that physically impact high-frequent resonators for energy generation^23,24^. Since the free sliding structures can effectively absorb energy from ambient vibrations, these kinds of FUC harvesters are suitable for electricity generation under wideband vibrations^21,23,24^.

Triboelectric flexible harvesters have recently been developed for generating energy from body movement, where free electrons can be induced by the rubbing between a dielectric material and an electrode^25–27^. Novel woven structures of triboelectric flexible harvesters have also been developed, which show great potential in large-scale and low-cost applications^28^. Based on a single-friction-surface scheme, transparent triboelectric generating sheets are very convenient for self-powered portable applications, where light human finger touching can induce considerable energy generation^29^. Moreover, the triboelectric generation technique has been widely used to convert wind energy, tide energy, etc. into electric power^30^.

In this study, a tiny wearable energy harvester is proposed and developed for efficiently generating stable energy from low-frequency limb movement. In the harvester, a micro-electroplated nickel ferromagnetic cantilever and a magnet are individually bonded on a flexible substrate, and in the initial state, they are attracted into contact. When the substrate is elongated owing to limb stretching, the cantilever releases from the magnet to resonate. When the substrate rebounds back due to limb retraction, the cantilever is pulled back to the magnet to form a clamped-supported beam that will resonate again in a higher resonance mode. With a piezoelectric thin film attached to the surface, the device can be excited into resonant generation twice within one limb movement cycle. Since the low-frequency horizontal stretching/rebounding movement of the substrate is frequency up-converted into a vertical high-frequency resonance, the harvester can freely resonate and generate stable electric power during each cycle of limb movement. The design, simulation, micro-fabrication, and wearable testing results of the novel flexible-substrate harvester will be detailed in the following.

## Materials and methods

### Structure design

**Fig. 1:**
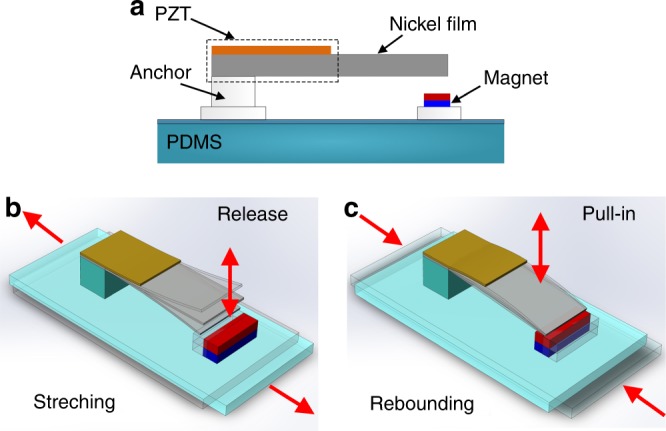
Structure and working principle. **a** Cross-sectional view of the energy harvester. **b** Schematic of the flexible-substrate stretching process that causes the cantilever release from the magnet and free resonance/generation at the up-conversion frequency. **c** Schematic of the flexible-substrate rebounding process that causes pull-in again of the cantilever end to generate electricity at the higher clamped-supported mode up-conversion frequency

Figure 1a shows a cross-sectional schematic of the proposed wearable energy harvester. An NdFeB magnet and a micromachined nickel cantilever are individually fixed by gluing them to a polydimethylsiloxane (PDMS) film, serving as a flexible substrate, via two glass pedestals. A lead-zirconate-titanate (PZT) piezoelectric ceramic film is bonded to the top surface of the cantilever, with conductive silver paste as the bonding media layer.

Figure 1b, c illustrate the FUC harvesting scheme within one elongation/rebounding cycle of the flexible substrate. In the initial state, the end of the ferromagnetic cantilever is pulled into contact with the magnet. When the PDMS substrate is stretched out, the piezoelectric cantilever will abruptly release from the magnet to cause free resonance/generation at its resonant frequency for a while, and then the resonance is attenuated to zero due to damping. When the substrate rebounds, the magnet will attract the cantilever again. When the magnetic force surpasses the restoring force of the cantilever, the magnetized nickel cantilever will impact and pull-in onto the magnet to form a beam with one end clamped and the other simply supported. Excited by the impact, the beam will resonate at a much higher resonant frequency than previously. The flexible device can be worn on a human joint, with both ends of the substrate adhered to the two sides of the joint. As the joint continuously rotates, the releasing/pull-in cycle will repeat. The horizontal stretching and the vertical resonance are perpendicular, and the slow movement and the high-speed resonance are disparate in frequency. In other words, the two kinetic behaviors cannot couple and influence each other. Therefore, the power generation efficiency can remain stable during one cycle. Based on this FUC generating scheme, the ultra-low frequency (e.g., sub-Hz to several Hz) limb movement can trigger a high-frequency efficient energy generating resonance at hundreds of Hz.

### Analysis and simulation

The two FUC processes are individually calculated, with the assumption that, during pull-in, the cantilever is changed to a clamped-supported beam that has one end fixed and the other simply supported. Using the Rayleigh approximation method, the vibration-wave mode that satisfies the boundary conditions can be assumed as

1 $$U_i = \cos \left( {\beta _ix/L} \right) - \cosh \left( {\beta _ix/L} \right) + q_i\left[ {\sin \left( {\beta _ix/L} \right) - \sinh \left( {\beta _ix/L} \right)} \right]\hskip-15pt$$

where *i* is the order number of the model. The first three modes (i.e., *i* = 1, 2, and 3) are included in the solution, as they can ensure the analysis precision^31^. For the resonant cantilever schematically shown in Fig. 1b, the coefficient *q*_*i*_ in Eq. (1) can be determined as

2 $$q_i = \frac{{\sin \left( {\beta _i} \right) - \sinh \left( {\beta _i} \right)}}{{\cos \left( {\beta _i} \right) + \cosh \left( {\beta _i} \right)}}$$

where *β*_1_ = 1.875, *β*_2_ = 4.694, and *β*_3_ = 7.855. In contrast, the *q*_*i*_ of the clamped-supported beam [shown in Fig. 1c] is

3 $$q_i = - \frac{{\cos \left( {\beta _i} \right) + \cosh \left( {\beta _i} \right)}}{{\sin \left( {\beta _i} \right) + \sinh \left( {\beta _i} \right)}}$$

where *β*_1_ = 3.927, *β*_2_ = 7.069, and *β*_3_ = 10.210. Supplementary Information details the deduction steps for both the mode shapes and the relevant coefficients. The mass and stiffness matrix can be defined as^32^

4 $${\boldsymbol{M}} = {\int}_{\!\!\!\!\!0}^L {m(x)U_{m}U_{n}{\mathrm{d}}x}$$

5 $${\boldsymbol{K}} = {\int}_{\!\!\!\!\!0}^L {YI(x)U{\prime\prime}_{m}U{\prime\prime}_{n}{\mathrm{d}}x}$$

where *m*(*x*) and *YI*(*x*) are the distributed density and the bending stiffness along the length direction, respectively^33^. The subscripts *m* and *n* stand for the order number of the model.

Using the Eigen-value problem formula

6 $$\left( {{\boldsymbol{K}} - \omega _i^2{\boldsymbol{M}}} \right){\boldsymbol{\xi }}_i = 0$$

where *ω*_*i*_ is the *i*th order angular frequency, and the practically behaved vibration modes *φ*_*i*_(*x*) can be calculated by superposing all the three assumed modes in

7 $$\varphi _i(x) = \mathop {\sum}\limits_{i = 0}^N {{\boldsymbol{\xi }}_iU_i}.$$

In practice, the piezoelectric film does not fully cover the whole substrate, which substantially influences the distributed stress and generated voltage. In this case, Eq. (7) can be used for precisely calculating *φ*_*i*_(*x*).

**Fig. 2:**
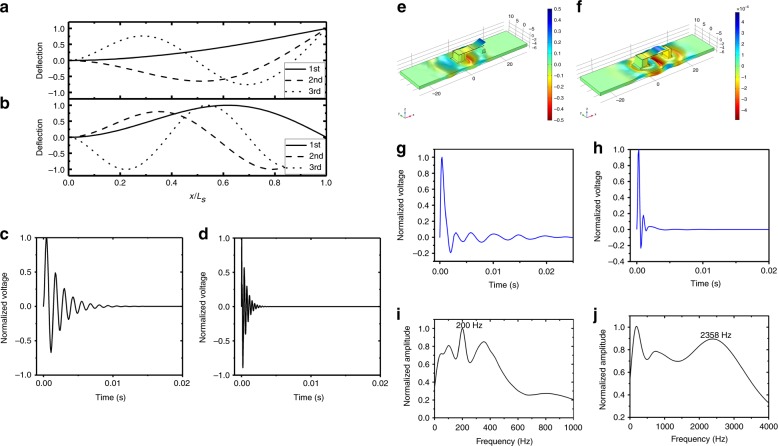
Analytical results of the actual first three resonant modes and the finite element simulation results of the two FUC processes of release and pull-in. **a** The cantilever and **b** the clamped-supported beam. The normalized time-domain output voltage after cantilever release is shown in (**c**), compared to that after pull-in shown in (**d**). Finite element simulation results for the release process are shown in terms of the (**e**) displacement, **f** normalized voltage waves, and **g** normalized voltage spectrum. For the pull-in process, the finite element simulation results are shown in terms of (**h**) displacement, **i** normalized voltage waves, and **j** normalized voltage spectrum

The first three modes of the cantilever and the beam are shown in Fig. 2a, b, respectively. The deflection of the two kinds of vibration structures can be solved using

8 $$u(t) = \mathop {\sum}\limits_{{m} = 0}^N {\varphi _{m}(x)r_{m}(t)}$$

Then, with *U*_*i*_ replaced by *φ*_*i*_, ***M*** and ***K*** in Eqs. (4) and (5) are diagonalized. After diagonalization, the electromechanical coupled dynamic equation and Kirchhoff’s law of the circuit loop can be used to establish an equivalent circuit model as

9 $$M_i\ddot r_i + D_i\dot r_i + K_ir_i - \theta _iv = F_i$$

10 $$\mathop {\sum}\limits_{i = 0}^N {\theta _i\dot r{}_i + C_{\mathrm{P}}\dot v + \frac{v}{{R_{\mathrm{L}}}}} \,{\mathrm{ = }}\,\,0$$

where *r*_*i*_, *M*_*i*_, *D*_*i*_, and *K*_*i*_ are the displacement, mass, damping coefficient, and stiffness of the *i*th mode diagonal components, respectively. *D*_*i*_ can be obtained using $D_i{\mathrm{ = }}2\zeta \sqrt {M_iK_i}$, where *ζ* is the damping ratio. *v* is the output voltage across load resistor *R*_L_. The capacitance *C*_P_ of the piezoelectric film can be calculated as $C_{\mathrm{p}} = bL_{\mathrm{p}}\varepsilon /h_{\mathrm{P}}$ . θ_*i*_ is the piezoelectric coupling factor, which can be deduced from the piezoelectric constitutive equation^29^. The material and geometric parameters of the piezoelectric cantilever are listed together in Table 1

**Table 1:** Material and geometric parameters of the device

Parameter	Symbol	Value
Thickness of the nickel cantilever film	*h*_s_	84 μm
Thickness of the piezoelectric film	*h*_p_	60 μm
Length of the nickel film	*L*_s_	11.0 mm
Length of the piezoelectric film	*L*_p_	5.0 mm
Width of the nickel and piezoelectric films	*b*	4.5 mm
Young’s modulus of nickel	*E*_s_	200 GPa
Young’s modulus of PZT	*E*_p_	53 GPa
Density of the nickel film	*ρ*_s_	7500 kg/m^3^
Density of the piezoelectric film	*ρ*_p_	8890 kg/m^3^
Damping ratio	*ζ*	0.1
Piezoelectric coupling	*d*_31_	−2.8e-10 m/V
Relative electrical permittivity	*ε*	4500
Residual magnetism of the magnet	*B*_r_	1 T
Size of the magnet	—	4 × 1.5 × 1.5 mm^3^
Size of the anchor	—	4 × 2 × 4 mm^3^

.

The normalized output voltage waves of the cantilever and the beam obtained using Eqs. (9) and (10) are shown in Fig. 2c, d, respectively. The low-frequency human movement is frequency up-converted into high-frequency resonant electricity.

Since the cantilever anchor on the flexible substrate is still compliant, the PDMS substrate somewhat influences the resonance of the cantilever. To obtain a more precise solution, the COMSOL finite element analysis software is used to simulate the complex vibration process, which includes the interaction between the flexible substrate and the stiff cantilever. Figure 2e shows the three-dimensional (3D) model for transient *z* axis displacement during the cantilever release. Figure 2f, g show the time-domain normalized voltage wave shape and the normalized frequency spectrum of the voltage during release. The observed peak at 200 Hz shows the resonant frequency of the cantilever. Similarly, Fig. 2h–j show the displacement, normalized voltage wave shape, and normalized frequency spectrum of the pull-in process. The much higher frequency peak at 2358 Hz corresponds to the resonance of the clamped-supported beam.

When the magnet is laid right under the cantilever, the magnetic force should surpass the restoring force of the cantilever such that the cantilever is attracted to the magnet. After the cantilever is attached to the magnet, the other parts of the magnetized cantilever structure have a magnetic interaction with the magnet. Fortunately, this magnetic interaction does not greatly influence the electric energy generation of the pull-in and release processes. Both the calculation and simulation show that, after the free end of the cantilever is attached to the magnet, the single-side clamped cantilever is changed to a clamped-supported beam, and its spring constant will increase by >45 times. In this case, the interfering magnetic interaction becomes much weaker compared to the stiffened spring restoring force; thereby, the influence on the resonant mode and frequency is very small. However, when the substrate is stretched, the magnet horizontally moves far from the end of the cantilever (shown in Fig. 1b), and the magnetic force exerted on the cantilever end will rapidly reduce to further weaken the aforementioned magnetic influence.

The space distance between the beam and the magnet should be small to ensure that the magnetic force can be larger than the restoring force of the cantilever. Under such a condition, the pull-in process and FUC electric generation can be realized. At the opposite extreme, the space should be large enough to ensure reliable release of the cantilever from attachment to the magnet. Additionally, the thickness of the cantilever anchor (almost equal to the space distance) should be thick enough to satisfy the mechanical clamping condition. After calculation and simulation, the 7740# Pyrex-glass anchor is designed with a 4 mm thickness and an area of 4 × 2 mm^2^, as shown in Fig. 1a.

### Fabrication

**Fig. 3:**
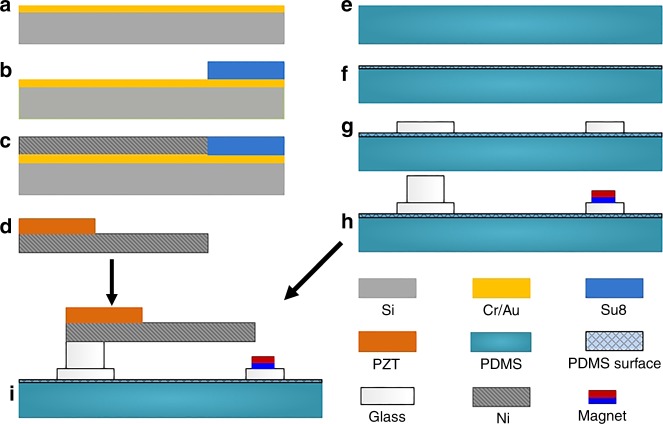
Fabrication process of the energy harvester. **a** A seed layer is sputtered on a wafer. **b** A photoresist layer is spin-coated and patterned. **c** A nickel film is electroplated. **d** The nickel film is released. Then, a PZT ceramic sheet is bonded. **e** A PDMS substrate is fabricated. **f** The PDMS is plasma treated. **g** Glass pedestals are attached on the PDMS. **h** Another glass block and a magnet are bonded on the two pedestals, respectively. **i** The piezoelectric cantilever is fixed on the glass block.

The fabrication flow chart shown in Fig. 3 will be detailed as follows.

- A Cr/Au layer (300/3000 Å) is sputtered onto a 4-inch silicon wafer to form the seed layer for the following micro-electroplating.
- After the adhesion promoter (OminCoat^TM^) is spin-coated, 90 μm-thick SU8-3050 photoresist is spin-coated and patterned using photolithography.
- Then an 84 μm-thick nickel film is micro-electroplated. The electroplating solution mainly consists of 300 g/l Ni(NH_2_SO_3_)_2_, 30 g/l NiCl_2_.6H_2_O and 30 g/l H_3_BO_3_. To form the nickel film with a low stress and a smooth surface, the electroplating conditions are set as follows: pH = 3.25, temperature = 50 °C, current density = 9 mA/cm^2^, and plating time of 22 h^34^.
- After removal of the SU8 photoresist, the substrate silicon is etched off using KOH with a 42% concentration at 85 °C to release the formed nickel film. Then conductive silver resin (H20E) is screen-printed on the surface of the nickel film. A PZT ceramic sheet (already with metal electrode films on the double sides) is bonded at the cantilever root to form the piezoelectric cantilever.
- A PDMS substrate (60 × 15 × 1.5 mm^3^) is fabricated by solidification in a mold and is then peeled out.
- The PDMS surface is modified using an oxygen-plasma treatment to enhance the bonding strength with the glass^35,36^.
- Two square glass pedestals are attached on the plasma-treated PDMS substrate to achieve spontaneous bonding.
- Another glass block and a small piece of NdFeB magnet are bonded on the glass pedestals using an ultraviolet (UV) light-assisted bonding glue.
- The piezoelectric cantilever is finally fixed on the glass block via the UV light-assisted bonding glue.

## Results and discussion

**Fig. 4:**
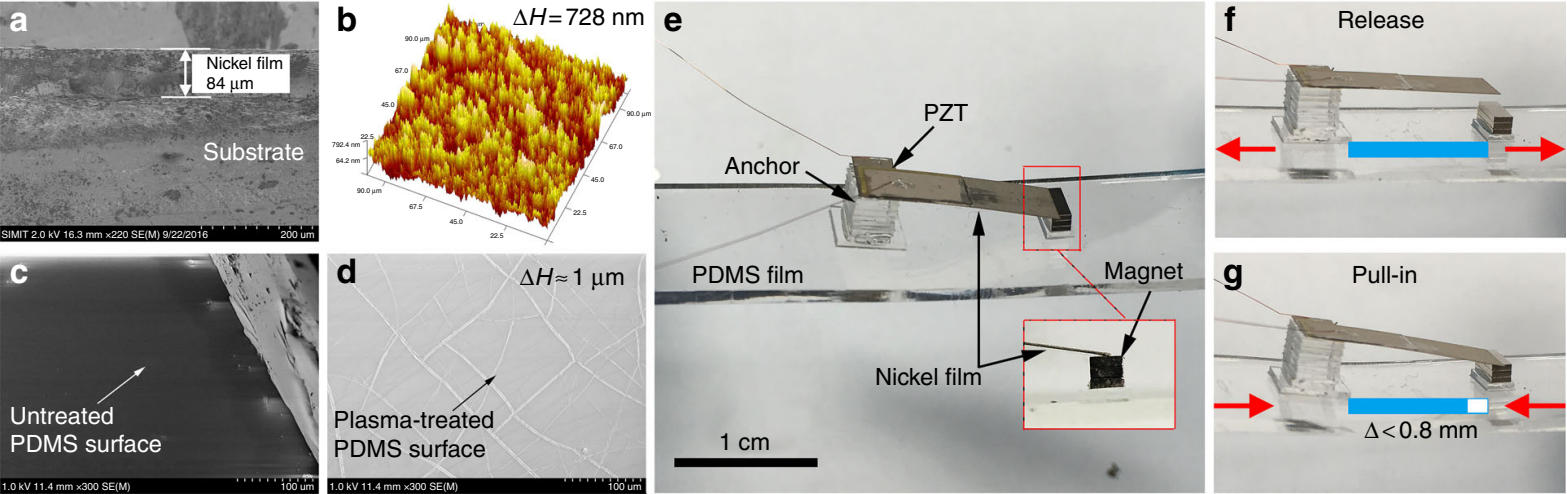
Fabricated energy harvester. **a** SEM image showing a cross-sectional view of the nickel film. **b** AFM scanned surface morphology of the nickel film. **c** SEM of the PDMS surface. **d** SEM of the PDMS surface after the plasma treatment. **e** Photograph of the fabricated energy harvester, with the inset showing the simple support by the magnet of the attracted cantilever end. **f**, **g** The release and pull-in states of the energy harvester

The scanning electron microscopic (SEM) image in Fig. 4a shows a cross-sectional view of the electroplated 84-μm-thick nickel film on the silicon substrate. Figure 4b shows the scanned surface morphology of the nickel film obtained using an atomic force microscope (AFM). The surface roughness is generally <0.8 μm. SEM images of the PDMS surface before and after the plasma treatment are compared in Fig. 4c, d. The plasma treatment can roughen the PDMS surface (roughness ≈ 1.0 μm) and generate high-density chemical groups for firm bonding with glass. The photograph in Fig. 4e shows the fabricated energy harvester. Figure 4f, g show the stretching and rebounding states of the energy harvester. By horizontally stretching the PDMS film, the change of the distance between the two glass pedestals is normally <0.8 mm.

**Fig. 5:**
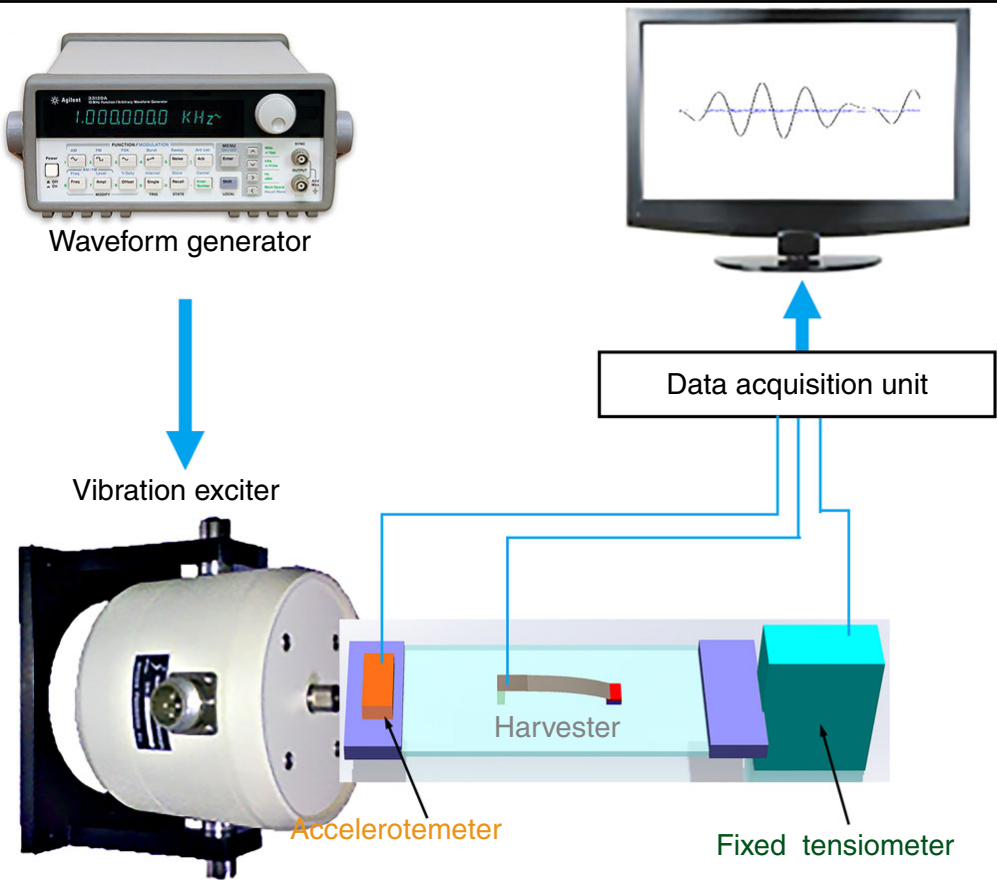
Schematic of the stretching test setup

The test setup is schematically shown in Fig. 5. A wave-form generator (Agilent 33120A) is used to excite a vibration shaker (JZK-5) to repeatedly stretch the flexible harvester. The two sides of the PDMS substrate are fixed on the shaker and a standard tensiometer. A standard accelerometer is also installed on the shaker to monitor the acceleration of the shaker movement. A data acquisition unit (NI USB-6003, 16 bit) is used to record the three transient signals from the accelerometer, the harvester, and the tensiometer. The harvester is loaded with a 40-kΩ matching resistor.

**Fig. 6:**
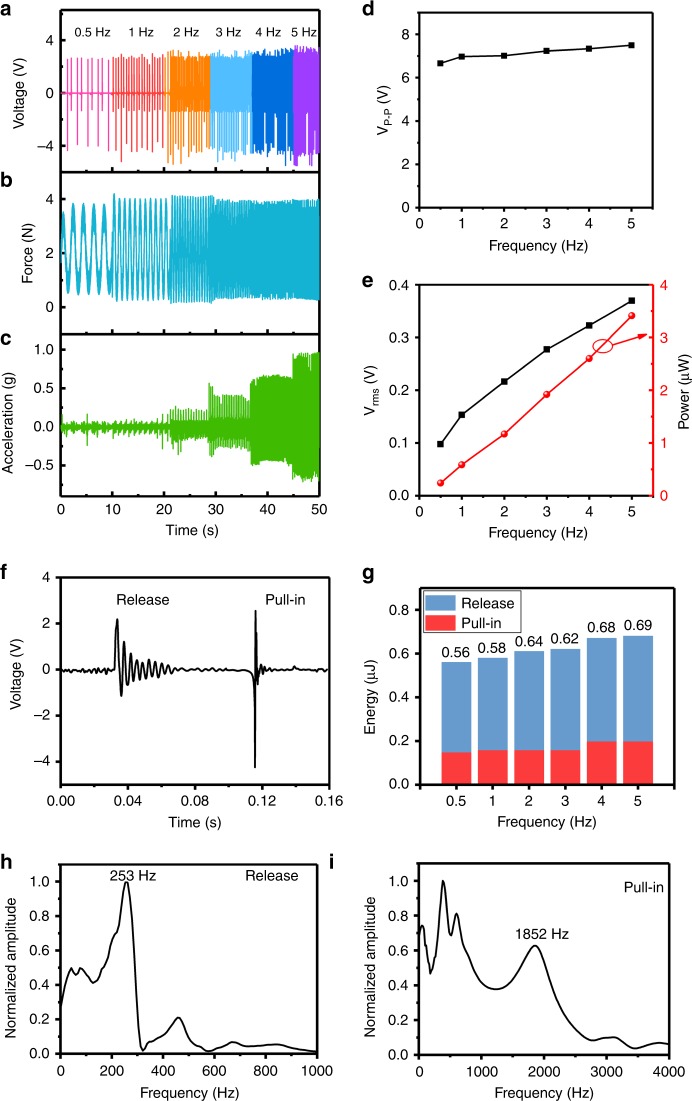
Test results. Time-domain test results of the generated voltage (**a**), stretching force (**b**), and stretching movement acceleration (**c**) for the stretching frequency range of 0.5–5.0 Hz. Frequency-domain test results of *V*_p-p_ (**d**), *V*_rms_, and power (**e**) for the frequency range of 0.5–5.0 Hz are also given. Test results for two voltage waves within one stretching cycle. **g** Frequency-dependent averaged electric energy in one movement cycle. The generated energy from the cantilever release process and pull-in process is separately indicated. **h**, **i** The normalized voltage spectrum for the release process and the pull-in process, respectively

Figure 6a shows the time-domain voltage generated under a series of low frequencies from 0.5 to 5.0 Hz. The stretching force and stretching movement acceleration are tested using the tensiometer and accelerometer, producing the results shown in Fig. 6b, c, respectively. To ensure the uniformity of the stretching-induced strain throughout the test, the maximum amplitude of the stretching force is kept as 2.0 N. The pre-stretch force of 2 N is designed to ensure that the PDMS film is always in the tensile state. The maximum acceleration is always kept at <1 × *g*, which corresponds with limb movements. Figure 6d shows the generated peak-to-peak voltage *V*_p-p_, which is always in the stable range from 7.5 V to 6.7 V when the frequency decreases from 5.0 Hz to 0.5 Hz. Figure 6e shows both the root-mean-square voltage (*V*_rms_) and the averaged power within the same frequency range. The relation of the power and frequency is quite linear, which indicates that identical energies are generated in one stretching/rebounding cycle. Our proposed wearable harvester can generate stable and efficient electric energy. Figure 6f shows the test result for voltage waves during one stretching/rebounding cycle, clearly demonstrating the two parts for the cantilever release process and the pull-in process. Figure 6g shows the relation between the averaged electric energy generated in one cycle and the stretching/rebounding movement frequency. In the whole frequency range from 0.5 Hz to 5.0 Hz, the generated energy within one movement cycle always remains in the stable range of 0.56–0.69 μJ. Taking the one-cycle generation of 0.69 μJ as an example, the generated average electric energy during release and pull-in is 0.48 and 0.21 μJ, respectively. Figure 6h is the normalized voltage spectrum for the release process. The peak at 253 Hz indicates the resonant frequency of the cantilever. Figure 6i shows the normalized voltage spectrum for the pull-in process. The peak near the much higher resonant frequency of 1852 Hz corresponds to the clamped-supported beam after pull-in. The testing results well verify the simulation results in Fig. 2g, j. The majority of the mechanical energy from the shaker vibration is absorbed by the PDMS to form the horizontal stretching. Only a very small part of the mechanical energy from the shaker is provided to the piezoelectric cantilever for vertical vibration and electric generation. Excluding the stretched PDMS substrate, the energy conversion efficiency of the cantilever–magnet interaction/generation structure is calculated to be approximately 9%. This device’s operation scheme is different from those of most inertial vibrating harvesters. Herein body-movement-induced horizontal substrate stretch is transformed into vertical vibration of the cantilever, i.e., the two movement directions are perpendicular. The cantilever longitudinal direction is along the in-plane direction of the structure for easy fabrication of the device chip. In future work, we will try to improve the device configuration to obtain a higher energy generation efficiency.

**Fig. 7:**
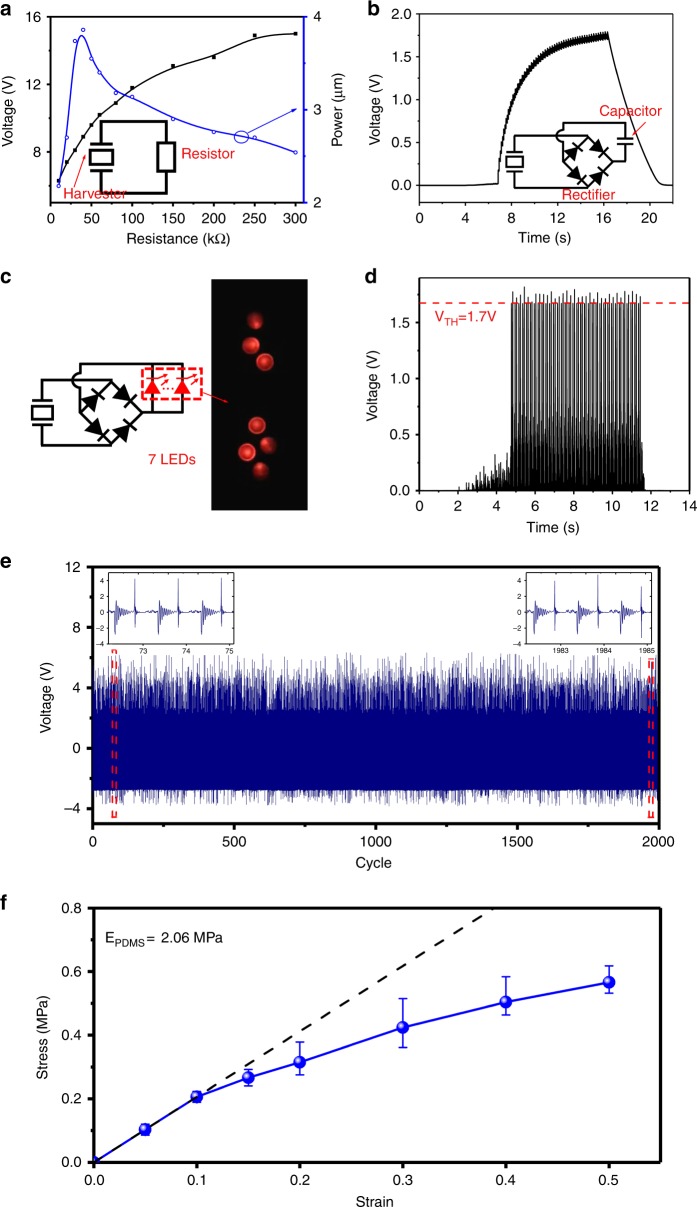
Test results for the harvester with various electric loads. **a** Generated *V*_p-p_ and averaged power vs. load resistance. **b** Time-domain voltage waves during the charging process of the harvester to a capacitor that is across a full-wave rectifier. **c** Seven red LEDs in parallel are lit by the harvester. **d** The voltage across the LEDs is quickly switched on, where the threshold voltage of the LED is approximately 1.7 V. **e** Test results for voltage waves during 2000 continuous cycles of stretching/rebounding. **f** Test results for the stress–stain relation of the PDMS substrate

Figure 7a–f show the voltage generation test results when the harvester is connected to different electrical loads. Herein the frequency is fixed as 5.0 Hz. Figure 7a shows the generated *V*_p-p_ and the average power for varied resistors. The matching resistor is 40 kΩ, which should be the same as the inner resistance of the harvester. The open-loop voltage is approximately 15 V. Figure 7b shows the charging/storage properties of the harvester, where a 2.1 μF tantalum capacitor is connected through a full-wave rectifier. After 5 s of charging, the voltage across the capacitor is 1.6 V, and the averaged power is 0.54 μW. Figure 7c shows that the stretched harvester successfully lights seven red light-emitting diodes (LEDs) in parallel. Figure 7d is the time-domain voltage across the LEDs, indicating that the harvester can quickly reach the 1.7 V threshold voltage of the LEDs to switch them on. The reliability of the device is also tested. Figure 7e shows the generated voltage waves during 2000 continuous cycles of stretching/rebounding of the flexible device. Since the generated power at the beginning and end of the test is 0.69 and 0.68 μJ, respectively, the change of the generated power is only 1.4%. Thus the generated power always remains stable throughout the test. Figure 7f shows the obtained relation of the stress and stain for the PDMS substrate obtained by testing the relation of the force and displacement. When the strain reaches the maximum of 0.5 (i.e., the length of the substrate increases 3 cm), no cracks occurs. As long as the strain does not exceed 0.1, which satisfies the requirement of most limb movements, the displacement of the substrate can be linear with the stretching force, and the Young’s modulus remains as 2.06 MPa.

**Fig. 8:**
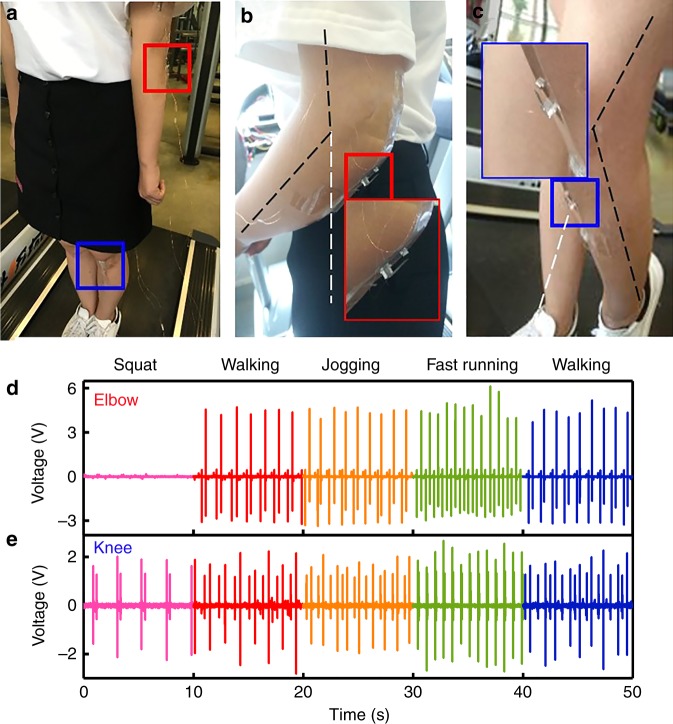
Wearable application. **a** Photograph showing a practical wearable application where two flexible harvesters are attached on a human elbow and knee. **b**, **c** Magnified views of the devices on the elbow and knee, respectively. **d**, **e** Test results for the voltage waves generated from the devices on the elbow and knee, respectively. The generated results are, sequentially, from joint bending (only for knee), walking, jogging, fast running, and walking again

As shown in Fig. 8a, two harvesters are individually attached to the elbow and knee of a person moving on a treadmill to demonstrate a practical wearable application. The magnified views of Fig. 8b, c show that the two wearable harvesters are mounted just under the joints of the elbow and the knee. For each harvester, the two ends of the PDMS substrate are taped to the two sides of the joint. Once the joint rotates, the device will undergo the cycled release/pull-in process. When the elbow or knee bends along with the limb’s movement, the substrates are stretched, and the harvesters are in the release state. When the two limbs return, the harvesters’ states are changed to pull-in. To ensure the occurrence of the two generation processes, the minimum bending angles of the elbow and the knee are 40° and 30°, respectively. In the experiment, two 40 kΩ matching resistors are connected to the two harvesters. The generated voltage waves for the harvesters at the elbow and the knee (both loaded with matching resistors) are shown in Fig. 8d, e, respectively. Typical limb movements, such as knee bending during a squat (once per 1.5 s.), walking (at the speed of 1-2 m/s), jogging (3–4 m/s), and fast running (5–6 m/s), are tested. A stable voltage can be always generated, with the device on the elbow generating a *V*_p-p_ of approximately 7.5 V and the device on the knee generating a *V*_p-p_ of approximately 4.0 V. The tested powers for different movements are listed in Table 2.

**Table 2:** Test results of the average power for different movements

Type of movement	Power (μW)	
	Elbow	Knee
Squatting	—	0.136
Walking	0.363	0.216
Jogging	0.389	0.226
Fast running	0.457	0.291

**Fig. 9:**
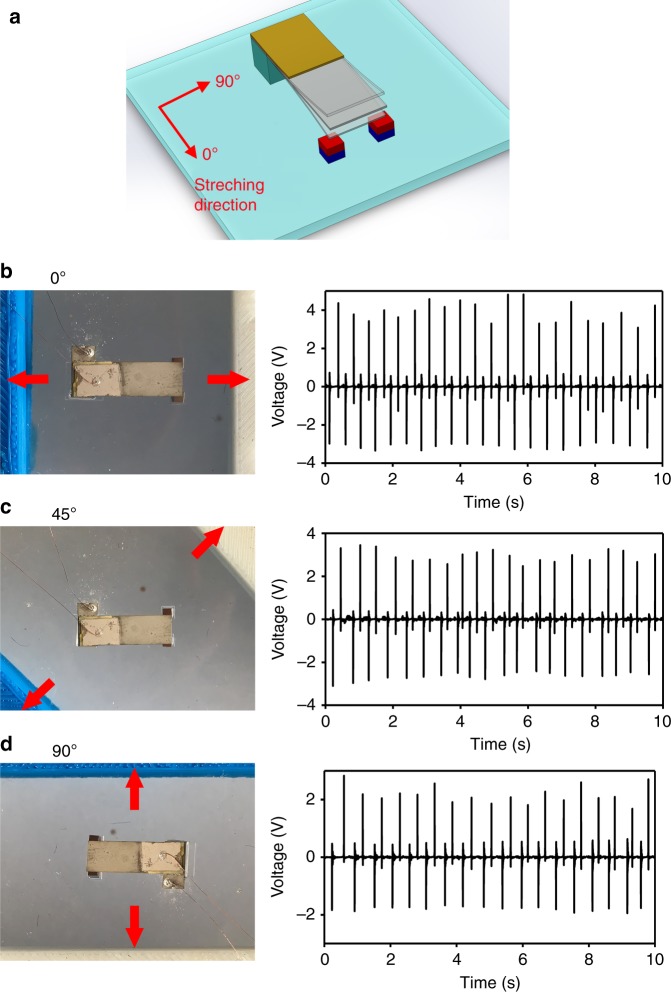
Two-directional energy harvester. **a** Schematic of the two-directional energy harvester for in-plane stretching along various directions. **b**–**d** When the stretching direction is along the device length direction, 45° inclined from the device length direction, and perpendicular to the device length direction, respectively; photographs of the harvester (on the left side) and the voltage waves generated during stretch (on the right side) are given

Moreover, the proposed FUC generating scheme can be used to achieve two-directional stretching energy harvesters, which are often required in wearable applications. Figure 9a schematically shows the 3D structure of a two-directional harvester, where two magnets are individually laid under the two vertex points of the cantilever free end. When the stretch is not along the cantilever length direction, the flexible substrate will be transversely lengthened, and the distance between the two magnets will be enlarged to cause the release of the cantilever. In this way, the harvester can generate electric energy regardless of what in-plane direction the external stretch is in. Figure 9b shows the fabricated two-directional harvester and the test results of the generated voltage waves when the stretch is along the cantilever length direction (indicated with the red arrow). Figure 9c, d are the voltage generation results for the stretch directions with deviations from the cantilever length direction of 45° and 90°, respectively. The generated *V*_p-p_ is stable and always >4 V for the three stretching directions, where the stretched displacement is unchanged.

Unlike most reported FUC harvesters, which usually transform the kinetic energy of inertial structures to electric power, the emphasis of the herein developed wearable FUC energy harvester is to transform horizontal low-frequency stretching movement into vertical high-frequency resonance for energy generation. Therefore, the device is suitable for usage with body movements, such as limb joint bending. The harvester demonstrates a good flexible-structure response to low-frequency movement and stable electric power generation within the per-cycle movement. Additionally, the achieved two-directional stretching energy harvesters in this study can potentially be attached to special clothes. However, it is difficult to weave the device into tissue structures. Currently, wearing the device by attaching the harvester to the opposite sides of a limb joint is somewhat inconvenient. In future work, we will try to improve the flexible-device packaging technique.

## CONCLUSION

This study proposes and develops a novel wearable energy harvester that frequency up-converts low-frequency human limb movements into the high-frequency electric generating resonance of a piezoelectric cantilever. Since the horizontal stretch of the flexible substrate has little influence on the vertical electric generating resonance, the harvester features stable electric energy generation in the sub-Hz to several Hz wide joint movement frequency range. In every limb movement cycle, the *V*_p-p_ and electric energy can remain stable even if the limb movement frequency changes within the range of sub-Hz to several Hz. The test results and human body wearable experiments have verified the usability of the flexible harvester. Additionally, a two-directional energy harvester for in-plane stretching in any direction is achieved using the same FUC scheme. The flexible-substrate harvesters have potential wearable applications.

## Electronic supplementary material

Supplementary Information of Wearable Energy-harvesters Generated from Low-frequency Human Limbs Movement
