# Supplementary material for: Wearable energy harvesters generating electricity from low-frequency human limb movement
Source: Microsyst Nanoeng. 2018 Sep 10;4:24. doi: 10.1038/s41378-018-0024-3 (PMC6220159; doi:10.1038/s41378-018-0024-3)
Supplement: Supplementary file 1 — Supplementary Information of Wearable Energy-harvesters Generated from Low-frequency Human Limbs Movement [file 41378_2018_24_MOESM1_ESM.docx]

Supplementary Information

of

**Wearable Energy-harvesters Generated from Low-frequency Human Limbs Movement**

Keli Li, Qisheng He, Jiachou Wang, Zhiguo Zhou, and Xinxin Li

The coefficients in Eq. (1) of $U_{i}=\cos\left(\beta_{i}x/L\right)-\cosh\left(\beta_{i}x/L\right)+q_{i}\left[\sin\left(\beta_{i}x/L\right)-\sinh\left(\beta_{i}x/L\right)\right]$ are obtained with the following deduction steps:

The transverse vibration equation of an Euler beam module is

$\frac{\partial^{2}}{\partial x^{2}}\left(YI\frac{\partial^{2}U}{\partial x^{2}}\right)+mS\frac{\partial^{2}U}{\partial t^{2}}=0$ (S1)

where *x* is longitudinal position on the beam, *U*, *YI*, *m* and *S* are mode shape, bending stiffness, density and sectional area of the piezoelectric beam, respectively. During the release process, the boundary condition of a resonant cantilever is

$U(0)=\frac{\partial U(0)}{\partial x}=\frac{\partial^{2}U(L)}{\partial x^{2}}=\frac{\partial^{3}U(L)}{\partial x^{3}}=0$ (S2)

with which Eq. (S1) can be solved. The principal mode can be obtained as

$U_{i}=\cos\left(\beta_{i}x/L\right)-\cosh\left(\beta_{i}x/L\right)+q_{i}\left[\sin\left(\beta_{i}x/L\right)-\sinh\left(\beta_{i}x/L\right)\right]$ (S3)

where *L* is length of beam. *i* is order number of mode. *β*_1_=1.875, *β*_2_=4.694 and *β*_3_=7.855. The coefficient *q_i_* in Eq. (S3) can be determined as

$q_{i}=\frac{\sin\left(\beta_{i}\right)-\sinh\left(\beta_{i}\right)}{\cos\left(\beta_{i}\right)+\cosh\left(\beta_{i}\right)}$ (S4)

During the pull-in process, the boundary condition of the fix-clamped/simple-supported beam is

$U(0)=\frac{\partial U(0)}{\partial x}=0$ (S5)

$U(L)=\frac{\partial^{2}U(L)}{\partial^{2}x}=0$ (S6)

Based on the boundary condition of Eqs. (S5) and (S6), the coefficient *q_i_* for the clamped-supported beam can be determined as

$q_{i}=-\frac{\cos\left(\beta_{i}\right)+\cosh\left(\beta_{i}\right)}{\sin\left(\beta_{i}\right)+\sinh\left(\beta_{i}\right)}$ (S7)

where *β*_1_=3.927, *β*_2_=7.069 and *β*_3_=10.210.
